# PP18 Red Flags For The Early Diagnosis Of Rare And Complex Connective Tissue And Musculoskeletal Diseases

**DOI:** 10.1017/S0266462324001910

**Published:** 2025-01-07

**Authors:** Guillermo Pérez García, Maria José Vicente Edo, Montserrat Salas Valero, Lucía Prieto Remón, Patricia Gavin Benavent

## Abstract

**Introduction:**

In collaboration with a European Reference Network for rare diseases, we aimed to identify red flags for the diagnosis of rare and complex connective tissue and musculoskeletal diseases (rCTDs). Some indicators, presented as red flags, might raise clinicians’ awareness about the presence of rCTDs. Their identification is critical in primary care, where they are most likely to be first observed.

**Methods:**

Firstly, we conducted a scoping review to identify red flags already published in the scientific literature. We included studies about people with rCTDs that described red flags, warning signs, alarm symptoms, and pathognomonic signs identifiable in a primary care setting. Then, we conducted a systematic review of evidence pointing out which signs and symptoms should arouse suspicion specifically for IgG4-related disease. We included studies providing estimates of diagnostic precision or prevalence of signs and symptoms, and we assessed their quality and applicability to the review question. We conducted systematic searches in major medical databases and manual searches in rare disease resources.

**Results:**

For the scoping review, 49 studies out of 1,656 records met the inclusion criteria. Two reported red flags for autoimmune diseases altogether, and 14 described red flags for systemic sclerosis. For the systematic review, seven studies out of 4,477 records met the criteria, comprising five diagnostic precision studies and two large case series. These were generally rated as having a high risk of bias and were included as indirect evidence. We identified 32 potential IgG4-related disease red flags, 10 related to clinical history findings and basic signs or symptoms, and eight belonging to common laboratory findings and basic imaging techniques.

**Conclusions:**

Red flags for rCTDs have generally been established through expert consensus and lack valid indicators for diagnosis, such as sensitivity, specificity, or predictive values. They frequently overlap among different rCTDs. Potential red flags are prone to change as further evidence emerges. This shows the need to collaborate with reference networks to address rare diseases where the evidence is still scarce.

